# The Association of Self-Reported Generalized Joint Hypermobility with pelvic girdle pain during pregnancy: a retrospective cohort study

**DOI:** 10.1186/s12891-020-03486-w

**Published:** 2020-07-20

**Authors:** Kerstin Ahlqvist, Elisabeth Krefting Bjelland, Ronnie Pingel, Angela Schlager, Lena Nilsson-Wikmar, Per Kristiansson

**Affiliations:** 1 Department of Public Health and Caring Sciences, Uppsala University, Husargatan 3, Box 564, 752 37 Uppsala, Sweden; 2 Department of Obstetrics and Gynecology, Akershus University Hospital, Lørenskog, Norway; 3 Department of Statistics, Uppsala University, Uppsala, Sweden; 4 Academic Primary Healthcare Centre, Stockholm County Council, Huddinge, Sweden; 5 Department of Neurobiology, Care Sciences and Society, Division of Physiotherapy, Karolinska Institutet, Huddinge, Sweden

**Keywords:** Generalized joint hypermobility, Pelvic girdle pain, Pregnancy, Five-part questionnaire, Pain drawing

## Abstract

**Background:**

Pelvic girdle pain (PGP) is common during pregnancy but the causes remain poorly understood. Generalized joint hypermobility (GJH) is an inherited trait, with joint mobility beyond normal limits and is assumed to be related with PGP. The aim of this project was to study the association between self-reported GJH and the presence of PGP during pregnancy.

**Methods:**

In this cohort study, 4884 Swedish-speaking women were consecutively recruited at their first visit for registration in the national antenatal screening programme in Sweden. We used the five-part questionnaire (5PQ) to assess GJH and pain drawings to identify PGP. Our primary outcome was the presence of PGP during the entire pregnancy and secondary outcomes were PGP in each trimesters. We tested the associations with logistic regression analysis, and adjusted for age and ethnicity.

**Results:**

In all**,** 2455 (50.3%) women responded to both questionnaires. The prevalence of self-reported GJH was 28.7%. A higher proportion of women with GJH than women without GJH reported PGP during the entire pregnancy (47.9% vs. 41.0%), particularly in trimester 1 (31.6% vs. 22.0%). Thus, women with GJH also had higher odds of PGP during the entire pregnancy (adjusted odds ratio (aOR) 1.27: 95% CI 1.11–1.47) and in trimester 1 (aOR 1.54: 95% CI 1.20–1.96), but the associations were not statistically significant in trimester 2 (aOR 1.24: 95% CI 0.82–1.88) or trimester 3 (aOR 1.20: 95% CI 0.99–1.45). The odds of PGP in pregnancy increased with increasing numbers of positive answers to the 5PQ (*p* for linear trend < 0.001) for the entire pregnancy and in trimester 1 (*p* for linear trend < 0.001), but not in trimesters 2 or 3 (*p* = 0.13 and *p* = 0.06, respectively).

**Conclusions:**

Compared to women with normal joint mobility, women with GJH had higher odds of reporting PGP during pregnancy and the odds increased with number of positive responses to the 5PQ. The associations were present in trimester 1 but did not reach statistical significance in trimester 2 and 3.

## Background

Worldwide, pelvic girdle pain (PGP) is a common condition during pregnancy [1] with a reported nine-month prevalence of 50% [2, 3]. PGP interferes with everyday life [4–7] and is a major cause of sick leave during pregnancy [8]. The aetiology of PGP remains uncertain but a high number of pregnancies, high body mass index (BMI), low age at menarche, physically demanding work and high levels of emotional distress have previously been reported as risk factors [9, 10]. Therefore, a multifactorial genesis has been proposed [2, 11, 12].

PGP typically debuts during the first half of pregnancy and regresses shortly after childbirth, indicating that pregnancy-related factors may affect structures in the pelvic area [4]. For instance, it is possible that changes in the connective tissues during pregnancy may play a role as decreased collagen turnover in early pregnancy has been associated with the development of PGP during pregnancy [13]. The pelvic joints may be more vulnerable to load and prone to pain development, especially in people with fragile connective tissue [14–16]. Constitutional weakness of the connective tissue may be reflected by increased peripheral joint mobility [17].

Previous studies have reported that joint mobility increases in the pelvic area and in peripheral joints during pregnancy and this increased joint mobility has been suggested to be one of the causes of PGP during pregnancy [17–19].

Generalized joint hypermobility (GJH) is a collagen phenotype that impacts the entire body [20–22]. GJH is defined as the capability of multiple joints,, to move beyond normal limits [23, 24]. GJH is a congenital and possibly inherited trait, which has similarities with heritable connective tissue disorders, such as fragile connective tissues [22, 23, 25]. Joint hypermobility decreases with age and is more prevalent in women and in some ethnic groups [26]. The prevalence of GJH also varies with the test and criteria used and with the population investigated, giving a reported prevalence of GJH varying between 2 and 57% [26–29]. GJH is estimated to occur in 10–20% in the general population [27].

The Beighton score (BeS), is the most commonly used method for clinical assessment of GJH [30] and a reliable tool for this purpose, but has shown shortcomings in studies on validity [29]. For questionnaire surveys, the self-reported five-part questionnaire (5PQ) is commonly used to identify past and present GJH [29, 31]. It was reported to be an effective method for identifying GJH when validated against BeS with a cut-off point of 4/9, providing sensitivity of 84% and a specificity of 80 and 89% (two cohorts) in the original study [31] and reported sensitivity of 70.9% and specificity of 77.4% in another study [32]. However, recent recommendations for measurement of GJH [29] and for the diagnosis of hypermobile Ehlers-Danlos syndrome [22] is a cut-off value of ≥5/9 on BeS for adults ≤50 years.

GJH is often asymptomatic but has also been associated with musculoskeletal pain [33, 34]. A recent study reported an association of GJH with chronic myofascial pelvic pain [35] but the association of GJH with PGP during pregnancy is scarcely investigated and conflicting results have been shown [14, 36]. We hypothesized that women with GJH would more often report PGP during pregnancy. The aim of this project was to study the association between self-reported GJH, measured using the 5PQ, and the presence of PGP, identified using pain drawings and reported for the entire pregnancy and in each trimester.

## Methods

This cohort study used self-reported data from the Swedish longitudinal pregnancy planning study (SWEPP). Information about the SWEPP study and an invitation to participate was distributed to all women who came to their first visit for registration in the national antenatal screening programme from September 2012 to July 2013. Materials and methods of the SWEPP study have been presented previously [37]. The SWEPP study is based on three questionnaires (Q1 - Q3) with responses preferably in Swedish, English or Arabic and no exclusion criteria. The present study used data from questionnaires (Q1 and Q2) in Swedish which had pain drawings available.

### Study population

All 196 antenatal clinics (ANCs) in nine Swedish counties, in central and in the northern Sweden, were invited to participate, with 144 clinics (73%) choosing to do so. ANCs were chosen to obtain a diverse range of socioeconomic characteristics among participants. Women who agreed to participate after receiving verbal and written information about the study, completed a questionnaire (Q1) at the ANC or at home and returned it in a prepaid envelope. A reminder was sent out 2 weeks after distribution.

**Fig. 1:**
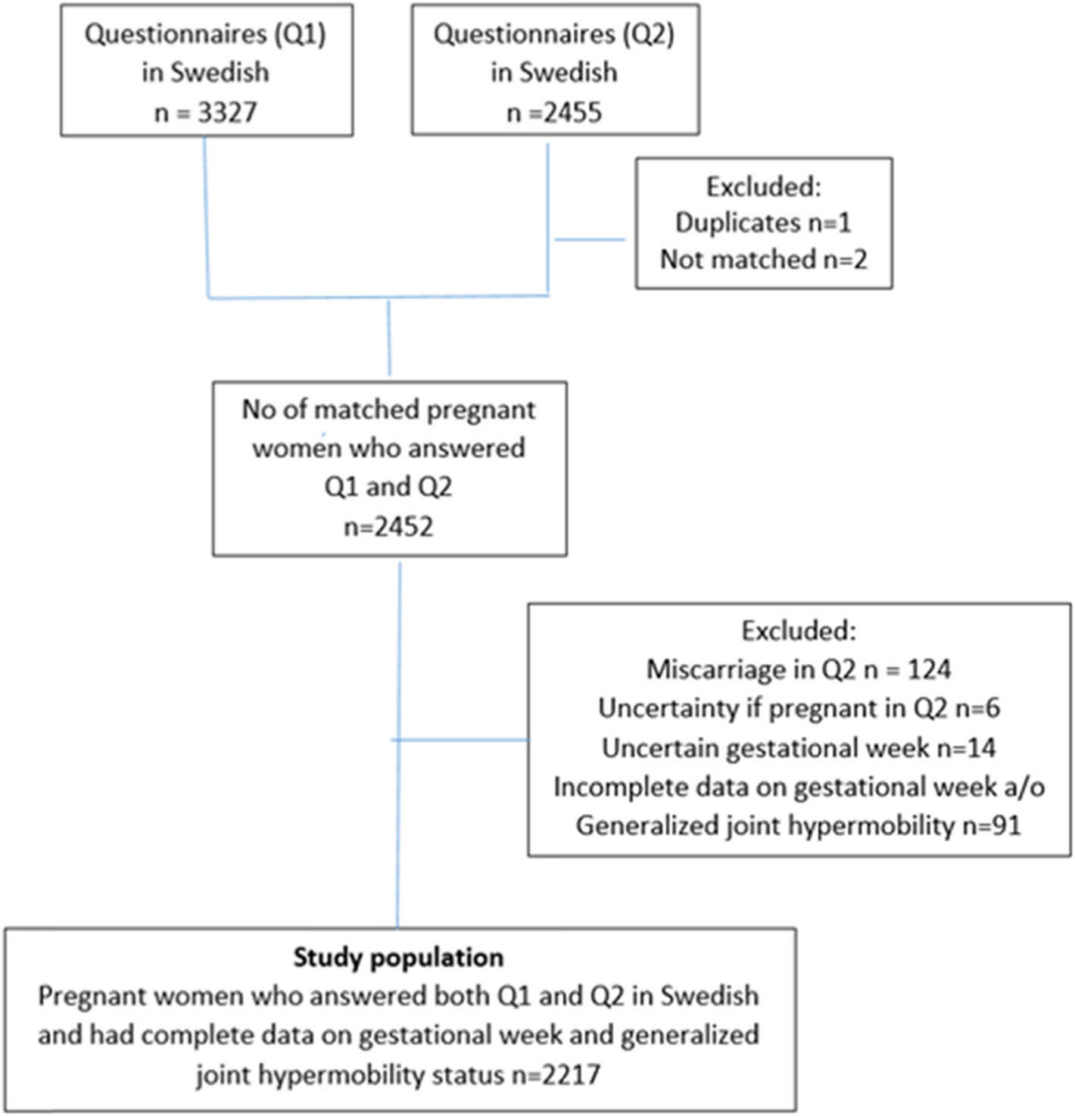
Data management of the Swedish questionnaires

In total, 5796 women were eligible for the SWEPP-study, 5493 women were approached and 4968 agreed to participate. The Swedish version of Q1 was delivered to 4884 women and answered by 3327 women (68%) responded. Of the women who responded to Q1, 3154 (65%) agreed to participate in the follow-up and 2455 (50%) responded to Q2 in gestational week (GW) 33–35 (estimated). After merging data from Q1 and Q2, 2452 women were matched. Of these, 235 women were excluded due to miscarriage (*n* = 124) or missing data on GW and/or GJH (*n* = 111), leaving 2217 women in our study population (Fig. 1).

### Data collection

Q1 was filled out in GW 11 (median range 3–36) and Q2 in GW 33 (median range 18–40). A total of 4434 questionnaires were filled out: 1656 during the first trimester (GW 0–12), 540 during the second trimester (GW 13–24) and 2238 during the third trimester (GW 25–40).

Q1 included questions about sociodemographic characteristics, general health and life-style. Q2 included the 5PQ, to identify women with GJH. Both Q1 and Q2 included a pain drawing, so respondents could indicate their pain locations. The pain areas indicated were manually transferred to a computer software program (Draw Survey, KLONK, Denmark) by two assistants, who were not part of the research team. For training purpose, 300 pain drawings were entered beforehand, using both computer mouse, roller mouse and touch pad, with the best results for computer mouse- and roller mouse, where the former was used in the study. To achieve the best possible transfer of the pain reports from paper to computer screen, the pain locations were entered using the anatomical landmarks on the drawings as reference points. All data input was double-checked to make sure that the transmission had been performed correctly, without yielding any pain locations beyond those reported on paper.

### Outcome measure

**Fig. 2:**
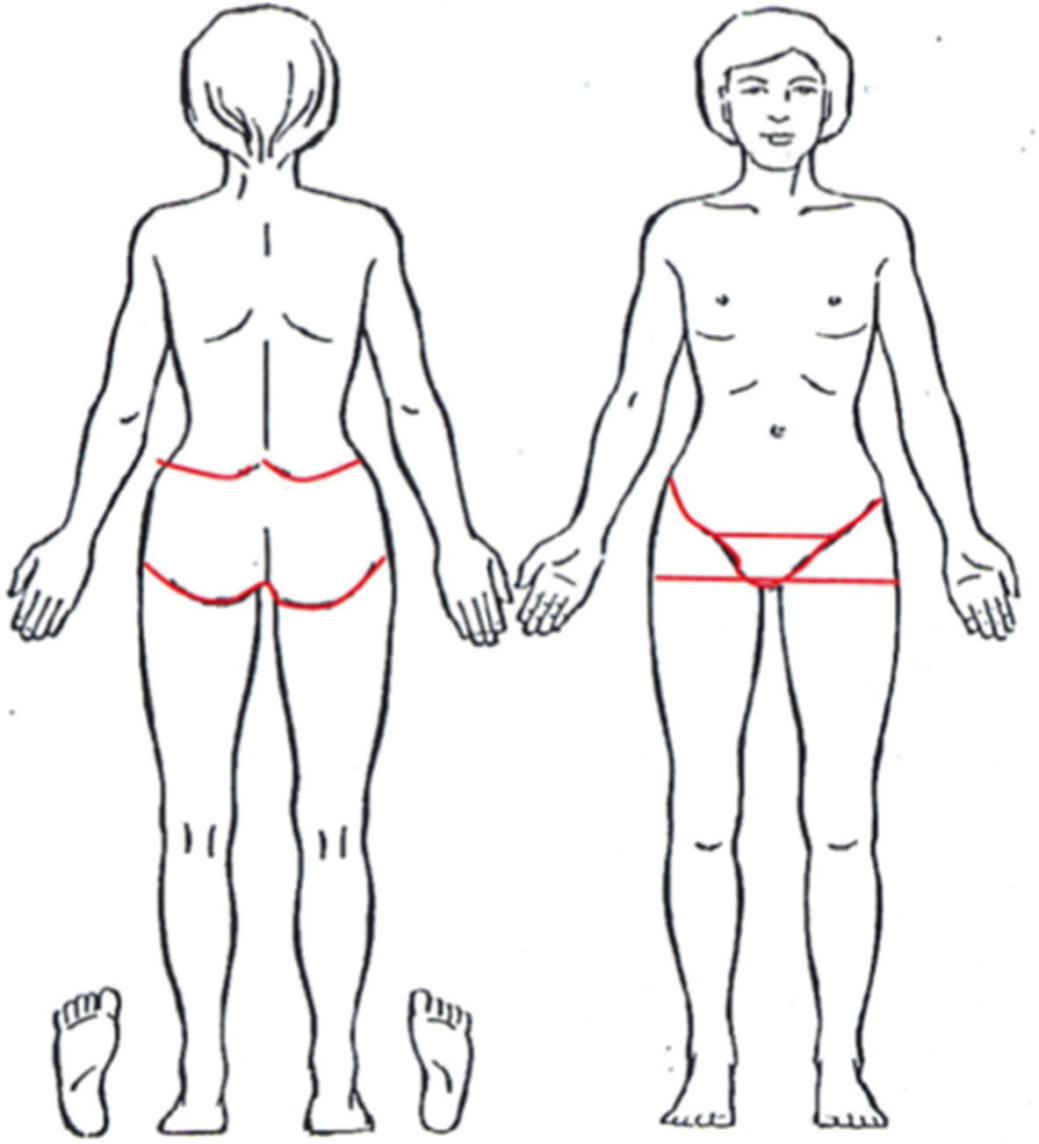
Drawing of a body to indicate pain locations. Pelvic girdle pain was defined when pain was indicated within the red boarders, not shown to the women

Our primary outcome measure was the presence of PGP, as indicated on a pain drawing, reported for the entire pregnancy. We used presence of PGP in each trimester of pregnancy as secondary outcomes. Pain between the posterior iliac crest and the gluteal fold and/or pain in the pubic symphysis or the groins was defined as PGP. The predetermined areas of the pelvic girdle region on the drawing, were not revealed to the women (Fig. 2).

### Exposure variable

Information about the presence of self-reported GJH was collected through the 5PQ [31]. The 5PQ consists of five questions: 1) “Can you now (or could you ever) place your hands flat on the floor without bending your knees?” (Yes/No), 2) “Can you now (or could you ever) bend your thumb to touch your forearm?” (Yes/No), 3) “As a child did you amuse your friends by contorting your body into strange shapes or could you do the splits?” (Yes/No), 4) “As a child or teenager did your shoulder or kneecap dislocate on more than one occasion?” (Yes/No) and 5) “Do you consider yourself double-jointed?” (Yes/No). Prior to the SWEPP study, the 5PQ was translated into Swedish from English and validity-tested by having it back-translated into Swedish by a native English translator. Each positive answer in the 5PQ yields 1 point, with a total score of 0–5. We used the cut-off point of ≥2 positive answers to define GJH. In additional analyses, we used the 5PQ scores categorized as: 0, 1, 2, 3 or 4–5 positive replies. The 5PQ has been reported to have good reproducibility and satisfactory sensitivity and specificity predicting joint hypermobility when BeS ≥4/9 [31].

### Covariates

We used information about maternal age, BMI (kg/m^2^), ethnicity with origin outside Europe, number of previous childbirths, education level, marital status and previous back pain.

### Statistical methods

Characteristics of the study sample are presented as means with standard deviation (SD) or medians with interquartile range (IQR) for continuous data and as numbers with proportions (%) for categorical data. Group differences were assessed using the two-sample t-test and Wilcoxon rank-sum test (Mann-Whitney *U*-test) for continuous data and test of proportions for categorical data. We used univariable and multivariable logistic regression analyses to test the association of GJH with presence of PGP during the entire pregnancy and in each of the trimesters, estimated as crude odds ratios (cOR) and adjusted odd ratios (aOR) with 95% confidence interval (95% CI). We adjusted for age and ethnicity after using directed acyclic graphs (DAGs). Given that some women had filled out Q1 and Q2 in the same trimester (4 women in trimester 2 and 22 women in trimester 3), we used the cluster robust standard error for the analyses of the entire period of pregnancy and in trimesters 2 and 3. Linear trends across number of positive responses to the 5PQ were tested by modelling joint hypermobility as a continuous variable. We performed additional analyses in strata of women on previously known risk factors [9, 10]. The risk factors were dichotomized for BMI (kg/m^2^) in trimester 1, “no overweight/obesity”: BMI = < 25.0 kg/m^2^ and “overweight/obesity”: BMI = > 25.0 kg/m^2^, parity (0/≥1), a history of back pain before the current pregnancy (yes/no) and physical activity 3 months before pregnancy (≥150 min/week, yes/no). To analyse drop-outs at Q2, we compared characteristics and pain at base-line between those that dropped out and those that remained. The prevalence of GJH was collected in Q2 and could therefore not be assessed in this analysis. Only two-tailed test were used. A 5% significance level was chosen for all analyses. All analyses were performed using STATA V.14.0 (Stata Corp, Texas USA). We followed the STROBE guidelines (http://www.strobe-statement.org).

### Ethical approval

The study was approved by the Regional Ethical Review Board in Uppsala, Sweden (2010/085).

## Results

### Characteristics

The mean age of the cohort was 29 years (SD 5 years) with a median BMI of 23.9 kg/m^2^ (IQR 21.9–27.3 kg/m^2^) and 55.8% reported at least one previous childbirth. Women with GJH more often had an origin outside Europe, had lower education levels and more often had experienced back pain before pregnancy compared with women without GJH (Table 1). The women who were lost to follow-up after Q1 (*n* = 874) more often had an origin outside Europe (14.7% vs 5.6%), more often were multiparous (74.6% vs 55.8%) and were less often university-educated (39.7% vs 50.1%) and 26.9% reported PGP in trimester 1 compared to 24.8% of the women who completed Q2 (data not shown).

**Table 1:** Baseline characteristics of the study population and stratified by self-reported generalized joint hypermobility

Variables	All women ***N*** = 2217 (100%)		Women with GJH ***N =*** 637 (28.7%)		Women without GJH ***N*** = 1580 (71.3%)	
	***n =***	n (%)	***n =***	n (%)	***n =***	n (%)
Age, years, mean (SD)^b^	1645	29.4 (4.8)	476	28.8 (4.9)	1169	29.6 (4.7)
BMI, median (IQR) kg/m^2,b^	1622	23.9 (21.9–27.3)	465	23.8 (21.9–26.8)	1157	23.9 (21.9–27.4)
Ethnicity, origin outside Europe, n (%)	2189	123 (5.6)	622	43 (6.9)	1567	80 (5.1)
Multiparous n (%)	2200	1228 (55.8)	630	361 (57.3)	1570	867 (55.2)
Completed university Education n (%)^a^	2217	1110 (50.1)	637	282 (44.3)	1580	828 (52.4)
Having a partner n (%)	2205	2177 (98.7)	629	621 (98.7)	1576	1556 (98.7)
History of back pain n (%)	2217	197 (8.9)	637	68 (10.7)	1580	129 (8.2)

*SD* Standard deviation, *IQR* Inter quartile range, *BMI* body mass index, *kg/m*^*2*^ kilograms per square meter, *GJH* Generalized Joint Hypermobility = sum score ≥ 2 in the five part questionnaire, *no GJH* sum score < 2 in the five part questionnaire

^a^ Statistical difference between women with and without GJH; *P <* 0.05

^b^ During first trimester

### Prevalence of self-reported GJH

The prevalence of self-reported GJH was 28.7% (*n* = 637) (Table 1). Among women classified with GJH, the questions “Can you now (or could you ever) place your hands flat on the floor without bending your knees?” and “As a child did you amuse your friends by contorting your body into strange shapes or could you do the split?”, were those most frequently answered positively, corresponding to 76.9% (*n* = 490) and 72.1% (*n* = 459), respectively.

### The association of GJH and PGP during pregnancy

The overall 9 month prevalence of PGP was 43% (Table 2). The proportions of women with PGP increased during pregnancy from 24.8% in trimester 1 to 30.9% in trimester 2 and 59.3% in trimester 3.

**Table 2:** The associations between self-reported generalized joint hypermobility and pelvic girdle pain in the 9-month period of pregnancy and in trimester 1, 2 and 3, expressed as crude and adjusted odds ratios with 95% confidence intervals

Pregnancy period: Entire Pregnancy Trimester 1–3 (***no of ques- tionnaires)***	All women	Women with GJH		Women without GJH		Crude OR (95% CI)	***P =***	Adjusted OR^**a**^ (95%CI)	***P =***
	Pain n (%)	Pain n (%)	No pain n (%)	Pain n (%)	No pain n (%)				
**Entire pregnancy** ***(n = 4434)***	1905 (43.0)	610 (47.9)	664 (52.1)	1295 (41.0)	1865 (59.0)	1.32 (1.15–1.52)	< 0.01	1.27 (1.11–1.47)	0.001
***1 (n = 1656)***	410 (24.8)	151 (31.6)	327 (68.4)	259 (22.0)	919 (78.0)	1.64 (1.29–2.08)	< 0.001	1.54 (1.20–1.96)	0.001
***2 (n = 540)***	167 (30.9)	53 (34.9)	99 (65.1)	114 (29.4)	274 (70.6)	1.29 (0.86–1.92)^b^	0.22	1.24 (0.82–1.88)^b^	0.30
***3 (n = 2238)***	1328 (59.3)	406 (63.0)	238 (37.0)	922 (57.8)	672 (42.2)	1.24 (1.03–1.50)^b^	0.02	1.20 (0.99–1.45)^b^	0.06

Pain was reported by completing pain drawings at two occasions during pregnancy

In total 2217 women filled in 4434 pain drawings of which 1656 were completed in trimester 1, 540 in trimester 2 and 2238 in trimester 3

*GJH* Generalized Joint hypermobility, sum score ≥ 2 in the five part questionnaire, *OR* odds ratio, *CI* confidence interval

^a^ Adjusted for age and ethnicity

^b^ Cluster robust standard errors

Women with GJH were more likely to report PGP during pregnancy than women without GJH (47.9% versus 41.0%, *p* = < 0.001). They were also more likely to report PGP in trimester 1 (31.6% versus 22.0%, *p* = < 0.01) and in trimester 3 (63.0% versus 57.8%, (*p* = 0.02), but not in trimester 2 (34.9% versus 29.4, *p* = 0.21 (Table 2).

The association between GJH and PGP for the entire pregnancy remained after adjustment for age and ethnicity (aOR 1.27: 95% CI 1.11–1.47). We also found an association of GJH with PGP in trimester 1 (aOR 1.54: 95% CI 1.20–1.96), which was smaller and non-significant in trimesters 2 and 3 (aOR 1.24: 95% CI 0.82–1.88; aOR 1.20: 95% CI 0.99–1.45) (Table 2). Additionally, the aOR of PGP in the entire pregnancy increased with increasing numbers of positive answers to the 5PQ (*p* for linear trend < 0.001) (Table 3) and in trimester 1 (*p* for linear trend < 0.001) but not in trimesters 2 or 3, (*p* = 0.13 and *p* = 0.06, respectively) (Table 4).

**Table 3:** The association of number of positive answers in the five part questionnaire with pelvic girdle pain in the 9-month period of pregnancy, among 2217 women reporting pain status two times during pregnancy (*n =* 4434 pain drawings)

Positive answers 5PQ	Pelvic girdle pain		
All women ***n =***	All cases n (%)	Adjusted OR^**a**^ (95% CI)	***P*** =
**Trimester 1**			
**0** 874	349 (39.9)	Reference	
**1** 706	299 (42.3)	1.10 (0.94–1.28)^c^	0.22
**2** 336	157 (46.7)	1.27 (1.05–1.53)^c^	0.02
**3** 202	98 (48.5)	1.36 (1.07–1.72)^c^	0.01
**4/5** 99	51 (51.5)	1.50 (1.08–2.10)^c^	0.02
**Test for linear trend**			≤0.001

*OR* odds ratio, *CI* confidence interval

^a^ Adjusted for age and ethnicity, ^b^ 5PQ – Five part Questionnaire: Generalized joint hypermobility = sum score ≥ 2

^c^ Cluster robust standard errors, *P <* 0.05

**Table 4:** The association of number of positive answers in the five part questionnaire with pelvic girdle pain in trimester 1, 2 and 3, among 2217 reporting pain status two times during pregnancy (*n =* 4434 pain drawings)

Positive answers 5PQ^**b**^	Pelvic girdle pain Trimester 1			Positive answers 5PQ^**b**^	Pelvic girdle pain Trimester 2			Positive answers 5PQ^**b**^	Pelvic girdle pain Trimester 3		
All women, ***n=***	All cases, n (%)	Adjusted OR^**a**^ (95% CI)	***P*** =	All Women ***n =***	All cases, n (%)	Adjusted OR^**a**^ (95% CI)	***P*** =	All Women ***n =***	All cases, n (%)	Adjusted OR^**a**^ (95% CI)	***P*** =
**0** 658	136 (20.7)	Reference		**0** 207	54 (26.1)	Reference		**0** 883	508 (57.5)	Reference	
**1** 520	123 (23.6)	1.20 (0.91–1.59)	0.20	**1** 181	60 (33.1)	1.33 (0.85–2.07)^c^	0.21	**1** 711	414 (58.2)	1.04 (0.85–1.27)^c^	0.73
**2** 252	73 (29.0)	1.49 (1.06–2.10)	0.02	**2** 80	29 (36.2)	1.59 (0.90–2.82)^c^	0.11	**2** 340	211 (62.1)	1.15 (0.89–1.50)^c^	0.29
**3** 152	52 (34.2)	1.96 (1.32–2.90)	0.001	**3** 48	12 (25.0)	0.75 (0.34–1.65)^c^	0.48	**3** 204	131 (64.2)	1.31 (0.94–1.82)^c^	0.10
**4/5** 74	26 (35.1)	1.75 (1.02–2.99)	0.04	**4/5** 24	12 (50.0)	2.78 (1.16–6.63)^c^	0.02	**4/5** 100	64 (64.0)	1.27 (0.82–1.95)^c^	0.29
	**Test for Linear trend**		≤0.001				0.13				0.06

^a^ Adjusted for age and ethnicity, ^b^ 5PQ – Five part Questionnaire: Generalized joint hypermobility = sum score ≥ 2, ^c^ Cluster robust standard errors, *P* < 0.05

The association of GJH with PGP displayed similar patterns in strata of women based on BMI in trimester 1, parity, a history of back pain and physical activity 3 months before pregnancy. However, the OR estimates for primiparous women, women with a history of back pain and women not physically active ≥150 min/week 3 months before pregnancy, did not reach statistical significance (Additional file 1).

## Discussion

Women with GJH had 27% higher odds of reporting PGP during the entire pregnancy period compared with women without GJH. The odds of PGP was 54% in trimester 1, but the association did not reach statistical significance in trimester 2 or trimester 3. The odds of PGP also increased with the number of positive responses to the 5PQ for the entire pregnancy and in trimester 1. The association between GJH and PGP during pregnancy further increased for women with multi parity and/or overweight.

Currently, the BeS is probably the most reliable method to assess GJH [29, 30]. Since this method is not suitable in questionnaire surveys, the prevalence of GJH in this study was measured using the 5PQ. It is easy to use and does not exclusively focus on five specific joints. The GJH prevalence of 28.7% was higher than we expected, compared with the estimated prevalence of 10–20% in the general population [27]. However, our prevalence is in accordance with that in a Danish survey of adults, which reported a prevalence of 30%, also using the 5PQ, with participants of both sexes and with higher mean age [38]. Our prevalence is also in accordance with a British survey using the 5PQ, for similar age group with both sexes included [39]. Farmer et al. reported a GJH prevalence of 17.6% in females, median age 39 years (range 18–89) based on BeS ≥4/9 and 5PQ ≥ 2 with a sensitivity and specificity of 0.85 [40]. On the contrary, a Brazilian study of university students reported a prevalence of 43.5% in female students using the 5PQ ≥ 2 and a prevalence of 44.3% using the BeS ≥4 [32]. This discrepancy in prevalence, may be due to that the participants in the Brazilian study were younger, most of them ranged from 17 to 24 years and the prevalence of GJH in non-Caucasians, is reported to be higher [27].

The prevalence of GJH in our study was based on the recommended cut-off point of ≥2 in the 5PQ [31]. However, the measurement properties for classifying GJH having been revised since the 5PQ was developed and the cut-off point for GJH having been raised from 4/9 to 5/9 of the BeS for women of fertile age [23, 29]. The increased cut-off point of the BeS may affect the optimal cut-off point of the 5PQ for in identifying GJH and thereby affecting the prevalence. If the 5PQ ≥ 2 falsely identified the women as having GJH, due to the revised cut-off point for the BeS, the association between GJH and PGP may be underestimated in the present study.

With a cut-off point of ≥3 in the 5PQ, the prevalence of self-reported GJH would be 13.6% in our study. Although a cut-off point of 2/5 in the 5PQ was suggested by Hakim et al. [31], our high prevalence of GJH may suggest that a higher cut-off score is needed for women of fertile age.

A recent Swedish study has validated the 5PQ ≥2 against BeS score of ≥5 for adults ≤50 years [41]. They reported a prevalence of GJH in females of 38.2% using the 5PQ and in the same study 24.7% using BeS ≥4 and 11.8% using BeS ≥5. Furthermore, they showed a sensitivity of 0.91, a specificity of 0.75%, low positive predicted value, high negative predictive value and the value of false positive rate (%) not reported.

Previous studies of the association between GJH and PGP during pregnancy are scarce with conflicting results [14, 36]. Comparison of the results is difficult as the instruments for assessing GJH and the diagnostic criteria for PGP differ. A cross-sectional study with retrospective data from 891 Swedish women found that women who reported being diagnosed with and/or had a family history of hypermobility had an increased risk of developing lumbar pain and/or PGP pain during pregnancy [14]. That study also relied on self-reported data, but it is unclear what measurement instrument they used to diagnose GJH. The prevalence of women diagnosed with GJH in that study (17.3%) like that in our study, is higher than the estimated prevalence of 10% in Western female populations [42, 43]. The higher prevalence of pain during pregnancy in that study, 72% vs 43% in our study, may be explained by the fact that they included lumbar pain in their outcome.

Another cross-sectional study reported no association between GJH and PGP during gestational weeks 13–40 [36]. They verified GJH with visually assessed BeS 5/9 and PGP was verified using pain provocation- and functional tests. The prevalence of GJH was 4.9% (*n* = 25). The lack of association could perhaps be explained by low statistical power. The prevalence of PGP (38%) was similar to that in the corresponding pregnancy period in our study.

Our hypothesis that women with GJH had an increased risk of reporting PGP during pregnancy was partly confirmed in the current study. Our findings showed an association between GJH and PGP during the nine-month pregnancy period. However, when the pregnancy period was divided into trimesters, the statistically significant association remained in trimester 1, but not in trimesters 2 or 3. The wide ranges of the response periods for Q1 (GW 3–36) and Q2 (GW 18–40) led to the results being presented in trimesters instead of as the beginning and end of pregnancy. The absence of a statistically significant association of GJH and PGP in trimester 2 may be due to low statistical power in the subgroup analysis.

The trend of PGP following the number of positive responses to the 5PQ in trimester 1 may indicate that women with GJH have fragile connective tissue, and may be more sensitive to the hormonal changes that occur in early pregnancy. Women with GJH reported PGP earlier in pregnancy than women without GJH in this study and it is known that there is increased sensitivity to chronic pain as a result of previous pain and pain duration [44, 45]. This might suggest that women with GJH could be at a higher risk of developing chronic pain postpartum.

The response rate of 50.3% in this study is similar to or higher than that in other questionnaire surveys [38, 39]. The women who dropped out after Q1 more often had an origin outside Europe and more often were multiparous compared with the women who completed Q2. Since these variables might have had an impact on both the exposure and the outcome, the drop-out may have introduced a selection bias and influenced the association between GJH and PGP. To reduce the risk of information bias, we excluded all questionnaires with incomplete data on GJH and GW since it affects the prevalence of PGP.

The strengths of this study include the longitudinal design and large study sample that generated 4434 pain drawings for analysis. Moreover, the use of pain drawings to identify PGP is a strength, as many studies do not distinguish between PGP and lumbar pain. We advocate using pain drawings instead of a single question to report PGP. For a person without any anatomical knowledge, it is likely easier to distinguish between the lumbar and the pelvic area on a drawing than answer a question about PGP. The pain drawing has been shown to be a reliable instrument to assess extent and location of acute and chronic pain [46, 47]. We also used a validated questionnaire (5PQ) for the exposure [31].

This study has limitations that require caution in the interpretation of the results. All data are based on self-report. The questions in the 5PQ may have been interpreted and answered differently by the participants and errors in reporting may have led to misclassification of GJH and biased our results.

Use of a pain drawing as an outcome measure has drawbacks. It is possible that the women in our study were misclassified when this method was used to identify PGP. People show a variety of behaviors when marking pain areas on a drawing. Some women may have marked their painful area very precisely, while others may have marked it widely, even outside the borders on the drawing. Because of the large study population, we believe that these phenomena did not cause a systematic bias between the study groups. Furthermore, transferring the pain drawings from paper to computer screen could have introduced a systemic bias since pain areas on paper are drawn slightly smaller [48] and the method has not been validated. However, since the size of the pain area would not be evaluated and the pain areas were double-checked by one of the two assistants responsible for the transformation and blinded for a presence of GJH or not in the assessed women, to ensure that the transmission had been performed correctly, without yielding any new pain locations, we anticipate that the risk of systematic bias is low.

Even though our response rate of 50.3% is similar or higher than in other questionnaire surveys [38, 39], it is a considerable amount of drop-outs that can introduce selection bias and influence the association between GJH and PGP. Since the exposure variable GJH was collected in Q2, we could not evaluate if women with GJH were more likely to drop out.

Future studies should be longitudinal, sufficiently powered and include reliable and valid measurement tools to assess GJH and PGP, in order to describe onset, fluctuations and persistence of PGP and evaluate an association between clinically assessed GJH and pregnancy-induced PGP during and after pregnancy.

### Implications

Pregnant women with pelvic girdle pain may benefit from being assessed for GJH early in pregnancy for preventive and therapeutic measures to avoid an increased risk of chronic PGP. The 5PQ may be used in a clinical setting as a quick screening instrument for GJH, followed by a more thorough clinical assessment to identify and diagnose GJH. However, future studies should investigate whether a cut-off point of ≥3 in the 5PQ is more optimal for identifying GJH in adults < 50 years.

## Conclusions

The prevalence of self-reported GJH was 28.7%. Women with self-reported GJH had higher odds for reporting PGP during the entire pregnancy period and in trimester 1, compared with women without self-reported GJH. The odds of PGP increased with the number of positive responses to the 5PQ. The associations of GJH with PGP in trimesters 2 and 3 were attenuated and did not reach statistical significance.

## Supplementary information

**Additional file 1.** The association between generalized joint hypermobility and pelvic girdle pain during pregnancy according to BMI, parity and a history of back pain, based on 4434 questionnaires from 2217 pregnant women.

## Data Availability

The datasets used and/or analyzed during the current study are available from the corresponding author on reasonable request.

## Abbreviations

PGP: Pelvic girdle pain
BMI: Body mass index
GJH: Generalized Joint Hypermobility
5PQ: 5-part self-reported questionnaire
SWEPP: Swedish longitudinal pregnancy planning study
Q: Questionnaire
GW: Gestational week
SD: Standard deviation
IQR: Interquartile range
cOR: Crude odd ratio
aOR: Adjusted odd ratio
DAG: directed acyclic graphs
LR: Likelihood-ratio
STROBE: Strengthening the Reporting of Observational Studies in Epidemiology.
