# Supplementary material for: The Association of Self-Reported Generalized Joint Hypermobility with pelvic girdle pain during pregnancy: a retrospective cohort study
Source: BMC Musculoskelet Disord. 2020 Jul 20;21:474. doi: 10.1186/s12891-020-03486-w (PMC7372850; doi:10.1186/s12891-020-03486-w)
Supplement: Supplementary file 1 — Additional file 1. The association between generalized joint hypermobility and pelvic girdle pain during pregnancy according to BMI, parity and a history of back pain, based on 4434 questionnaires from 2217 pregnant women. [file 12891_2020_3486_MOESM1_ESM.docx]

Additional file 1. The association between generalized joint hypermobility and pelvic girdle pain during pregnancy according to BMI, parity and a history of back pain, based on 4434 questionnaires from 2217 pregnant women.

|  | **No of Questionnaires** | **Crude OR**  **(95% CI)** | **Adjusted OR ^a^**  **(95%CI)** |
| --- | --- | --- | --- |
|  |  |  |  |
| **Women with BMI ^b^ <25** | 976 |  |  |
| Women with no GJH | 692 | Reference | Reference |
| Women with GJH | 284 | 1.57 (1.13-2.17) | 1.49 (1.06-2.08) |
| **Women with BMI ^b^ ≥25** | 646 |  |  |
| Women with no GJH | 465 | Reference | Reference |
| Women with GJH | 181 | 1.77 (1.23-2.55) | 1.62 (1.11-2.34) |
| **Primiparous women** | 1944 |  |  |
| Women with no GJH | 1406 | Reference | Reference |
| Women with GJH | 538 | 1.23 (0.99-1.53) | 1.18 (0.94-1.47) |
| **Multiparous women** | 2456 |  |  |
| Women with no GJH | 1734 | Reference | Reference |
| Women with GJH | 722 | 1.40 (1.16-1.68) | 1.35 (1.12-1.63) |
| **Women with no history of back pain** | 4040 |  |  |
| Women with no GJH | 2902 | Reference | Reference |
| Women with GJH | 1138 | 1.27 (1.09-1.46) | 1.22 (1.05-1.42) |
| **Women with a history of back pain** | 394 |  |  |
| Women with no GJH | 258 | Reference | Reference |
| Women with GJH | 136 | 1.66 (1.02-2.71) | 1.57 (0.97-2.56) |
| **Women not physically**  **active≥150 min/week**  **pre-pregnancy**  Women with no GJH  Women with GJH | 1942  1428  514 | Reference  1.17 (0.94-1.46) | Reference  1.13 (0.91-1.41) |
| **Women physically active ≥150 min/week**  **pre-pregnancy**  Women with no GJH  Women withGJH | 2446  1696  750 | Reference  1.48 (1.23-1.77) | Reference  1.41 (1.17-1.70) |

BMI ^b^, body mass index in trimester 1; CI, confidence interval; OR, odds ratio

^a^ Adjusted for age and ethnicity,
